# The burden of hypertension in the emergency department and linkage to care: A prospective cohort study in Tanzania

**DOI:** 10.1371/journal.pone.0211287

**Published:** 2019-01-25

**Authors:** Sophie W. Galson, John W. Stanifer, Julian T. Hertz, Gloria Temu, Nathan Thielman, Temitope Gafaar, Catherine A. Staton

**Affiliations:** 1 Department of Surgery, Division of Emergency Medicine, Duke University, Durham, North Carolina, United States of America; 2 Duke Global Health Institute, Duke University, Durham, North Carolina, United States of America; 3 Department of Medicine, Division of Nephrology, Duke University, Durham, North Carolina, United States of America; 4 Department of Medicine, Kilimanjaro Christian Medical College Hospital, Kilimanjaro, Tanzania; 5 Department of Medicine, Duke University, Durham, North Carolina, United States of America; 6 Department of Neurosurgery, Division of Global Neurosurgery and Neuroscience, Durham, North Carolina, United States of America; Purdue University, UNITED STATES

## Abstract

**Objectives:**

Globally, hypertension affects one billion people and disproportionately burdens low-and middle-income countries. Despite the high disease burden in sub-Saharan Africa, optimal care models for diagnosing and treating hypertension have not been established. Emergency departments (EDs) are frequently the first biomedical healthcare contact for many people in the region. ED encounters may offer a unique opportunity for identifying high risk patients and linking them to care.

**Methods:**

Between July 2017 and March 2018, we conducted a prospective cohort study among patients presenting to a tertiary care ED in northern Tanzania. We recruited adult patients with a triage blood pressure ≥ 140/90 mmHg in order to screen for hypertension. We explored knowledge, attitudes and practices for hypertension using a questionnaire, and assessed factors associated with successful follow-up. Hypertension was defined as a single blood pressure measurement ≥ 160/100 mmHg or a three-time average of ≥ 140/90 mmHg. Uncontrolled hypertension was defined as a three-time average measurement of ≥ 160/100 mmHg. Successful follow-up was defined as seeing an outpatient provider within one month of the ED visit.

**Results:**

We enrolled 598 adults (mean age 59.6 years), of whom 539 (90.1%) completed the study. The majority (78.6%) of participants were aware of having hypertension. Many (223; 37.2%) had uncontrolled hypertension. Overall, only 236 (43.8%) of participants successfully followed-up within one month. Successful follow-up was associated with a greater understanding that hypertension requires lifelong treatment (RR 1.11; 95% CI 1.03,1.21) and inversely associated with greater anxiety about the future (RR 0.80; 95% CI 0.64,0.99).

**Conclusion:**

In a northern Tanzanian tertiary care ED, the burden of hypertension is high, with few patients receiving optimal outpatient care follow-up. Multi-disciplinary strategies are needed to improve linkage to care for high-risk patients from ED settings.

## Introduction

Cardiovascular disease is the leading cause of global morbidity and mortality [1, 2]. Sub-Saharan Africa (SSA) is particularly vulnerable to the growing global burden of cardiovascular disease, which disportionately impacts low-and-middle income countries (LMICs)[3]. Among the risk factors for cardiovascular disease, hypertension is the most pervasive and contributes the greatest attributable risk to overall cardiovascular death [4]. Currently, the prevalence of hypertension in SSA is estimated to to be as high as 40%, with fewer than 10% of affected individuals receiving optimal blood pressure control [5, 6]. Even more alarming, by the year 2025, as many as 125 million people in SSA will have hypertension [7]; yet, the region is starkly underprepared to address this growing public health crisis [8].

Over the past few decades, in an effort to build a sustainable health financing structure for non-communicable diseases such as hypertension, many SSA countries have made crucial changes to expand health insurance coverage and promote outpatient care [9]. Specifically in Tanzania, the National Health Insurance Fund (NHIF) was started in 2001 and is the most comprehensive publicly-owned health insurance scheme in the country [10]. Community Health Fund (CHF) and Tiba Kwa Kadi (TIKA) are other publicly-owned insurance strategies; they target the informal sector and are managed at district and municipal levels respectively [11]. However, although insurance coverage has steadily increased over the years, 80% of the population remains uninsured [9]. Per Tanzania’s national health policy, outpatient health centers (village health services, dispensaries, health centers, and district hospitals) are designated to manage ostensibly uncomplicated conditions, such as hypertension [12]. However, a recent study assessed the preparedness of 725 health facilities and found that only 28% were prepared for outpatient screening or primary care management of hypertension and many of these facilities lacked basic equipment such as blood pressure cuffs or treatment guidelines [13].

In locations like Tanzania, where the existing primary care system is poorly-equipped to manage the expanding hypertensive population, patients may ultimately end up seeking care in emergency departments (EDs) and inpatient settings. Several studies in high-income countries have established that the emergency department (ED) is an important venue to screen for hypertension, in part because a sustained elevated blood pressure in the ED is highly sensitive for hypertension [14–17]. However, it is unclear what role the ED could play in facilitating screening and initiating care for hypertension in LMICs, particularly in SSA [18]. In SSA, ED care is expensive however it is often the first point of healthcare contact for many people, and occasionally the sole source of medical care, excluding traditional healers [6, 19, 20]. Therefore, the ED may be one of many potential sites to screen patients, educate them about their disease, and ensure linkage to outpatient follow-up care [17, 21]. However, in SSA, even preliminary epidemiological data concerning the burden of hypertension and linkage to care from the ED are severely lacking.

Therefore, we conducted a study to determine the burden of elevated blood pressure in the ED and to explore the pathways of care in a high-risk population in Moshi, Tanzania.

## Materials and methods

### Ethics

The study protocol was approved by Duke University Institutional Review Board (#Pro00081220), the Kilimanjaro Christian Medical College (KCMC) Ethics Committee (EC#502), and the National Institute for Medical Research in Tanzania. Written informed consent was obtained from all participants, and all participants with abnormal findings received information about their results and were instructed to follow up with an outpatient provider within two weeks to one month which is the current standard of care.

### Setting

The study was conducted between July 2017 and March 2018 in the ED of KCMC, which is located in Moshi, a town in the Kilimanjaro region (population greater than 900,000) of northern Tanzania [22]. The largest ethnic group is Chagga and the primary language spoken is Swahili [22, 23]. Most residents have a primary education and the unemployment rate is 19% [23]. As of 2014, the local, community prevalence of diabetes was 5.7% and prevalence of obesity/overweight BMI was 58% [24].

KCMC is the referral hospital for northern Tanzania with over 630 beds and a regional training center [25]. It serves a population of over 11 million people [25] and the ED receives an average of 90 patients per day, the majority of whom arrive during the daytime and evening shifts (8AM to 11PM) (KCMC Traumatic Brain Injury Registry, unpublished results, Staton 2017).

All patients presenting to the ED are initially evaluated by a triage nurse and have their vital signs measured and recorded. Per the current head of emergency medicine at KCMC, all patients with elevated blood pressure who are discharged from the ED or the hospitial are instructed to follow-up in two weeks–one month with an outpatient provider.

### Sampling and data collection

#### Participant recruitment

We recruited participants using trained research nurses who were bilingual Tanzanian natives. Inclusion criteria were: adults (age ≥ 18 years old) with a measured initial triage systolic blood pressure ≥ 140 or diastolic blood pressure ≥ 90 mmHg. All adults presenting to the ED during the morning or evening shifts (8AM to 11PM) were recruited. Pregnant females (measured by urine human chorionic gonadotropin) and adults unable to be surveyed due to the severity of their illness or injury were excluded.

The primary study outcome was defined as self-reported attendance at an outpatient provider within one month following discharge from initial ED visit. We estimated that a sample size of 600 would detect a 10% difference in proportions (assuming 95% power and precision of 0.05) between the follow-up and non-follow-up cohorts. We anticipated a 40% loss to follow-up rate based on previous studies in this setting [26].

Initially, we included a pilot week of night shifts with a plan to stop if overall patient volume was low. Due to very low total patient volume at night (one patient recruited over one week), and study budget restraints, the night shifts were discontinued.

#### Survey

After informed consent, study participants were verbally administered a demographic and medical history questionnaire. The questionnaire was initially written in English and translated for administration in the local language of Swahili. A bilingual trained research nurse performed back translation of the questionnaire to verify content and review of the qualitative responses. Information gathered included age, gender, level of education, traditional medicine use, alcohol use, and tobacco use. If participants were receiving anti-hypertensive therapy, then specific drug information was collected. Women additionally gave information regarding pregnancy and menstruation history. Participants also completed a quantitative knowledge, attitudes, and practices (KAP) survey on hypertension. All instruments were adapted using previously validated surveys which were developed in Moshi, Tanzania, for chronic disease [27, 28].

#### Blood pressure measurement and urinalysis

The research nurses measured blood pressure using an automated Omron HEM-712 sphygmomanometer (Omron Healthcare, Inc.; Bannockburn, IL), which has an adjustable cuff size. The machine was calibrated monthly during data collection. All participants were seated in an erect position with feet flat on the floor for a minimum of five minutes before measurements were obtained, per World Health Organization (WHO) protocol [7]. Initial triage blood pressure was recorded and repeat blood pressure measurements were taken 60 minutes later [16]. Two blood pressure measurements were obtained at the second time-point, and the average of the three measurements was calculated according to WHO recommendations [7].

Hypertension was defined as a single systolic blood pressure measurement ≥ 160 mmHg, diastolic measurement ≥ 100 mmHg, or a three-time average measurement of ≥ 140 mmHg systolic or ≥ 90 mmHg diastolic, irrespective of treatment status. Uncontrolled hypertension was defined as a three-time average measurement of systolic ≥ 160 or diastolic ≥ 100 mmHg, irrespective of treatment status. Blood pressure definitions were chosen based on Tanzanian and Joint National Commision guidelines [12]. Hypertension awareness was defined as giving a self-reported disease history and subsequently testing positive in the screening process. Prevalence of hypertension was determined using data on total patients presenting to the ED. Data on patients presenting for anti-hypertension medication refills only were taken from the physician log.

A measure of end-organ damage was performed using a point of care urine dipstick to assess for proteinuria [29]. A mid-stream, clean catch urine specimen was obtained for analysis with Mission 10 Parameter Urinalysis test strips (ACON Labs, San Diego, CA). Proteinuria was defined semi-qualitatively as ≥1+ proteinuria [29].

### Study variables

To assess the primary outcome (self-reported attendance at an outpatient provider within 1 month following ED discharge), we made a 1-month post-ED follow-up phone call. At that time, patients were also asked to report any other forms of healthcare (e.g. traditional medicine provider) and all current anti-hypertensive medications. We did not specifically ask what type of provider (physician, mid-level, nurse, or pharmacist) was seen. A return visit to the ED did not qualify as an outpatient follow-up visit. To reduce non-response rates, we attempted a minimum of five additional phone calls during off-hours (evenings and weekends). When possible, data were triangulated with medical records to ensure accuracy.

### Data management

Data were collected on paper forms and entered into Redcap by a trained data entry technician [30]. Approximately 10% of all data were verified after electronic data entry by an independent reviewer to ensure accuracy.

### Data analysis

The mean and standard deviation (SD) were reported for continuous variables. Alternatively, when distributions were skewed, medians and inter-quartile ranges (IQRs) were used. Differences in likelihood of follow-up for hypertension were compared using chi-square or Fisher’s exact test for categorical variables with cell counts with fewer than 5 expected observations. Prevalence was estimated by dividing the total number of hypertensive patients by the total number of ED patients.

To explore associations between hypertension risk factors and predictors of follow-up after ED visit, crude and adjusted risk ratios (RR) were estimated using generalized linear models with a log link. Separate regression models to make adjusted bivariate comparisons were fitted to follow-up status for each lifestyle-related variable, including alcohol use, tobacco use, traditional medicine use, and current antihypertensive use. Separate regression models were also fitted with responses to all KAP questions regarding patient knowledge, attitudes, and practices. All models were adjusted for confounding factors potentially associated with hypertension, including age, gender, and ethnicity. We did not include education or occupation in our models due to *a priori* assumptions about potential upstream causal association with lifestyle-related risk factors.

## Results

### Prevalence of hypertension in the study population

**Fig 1:**
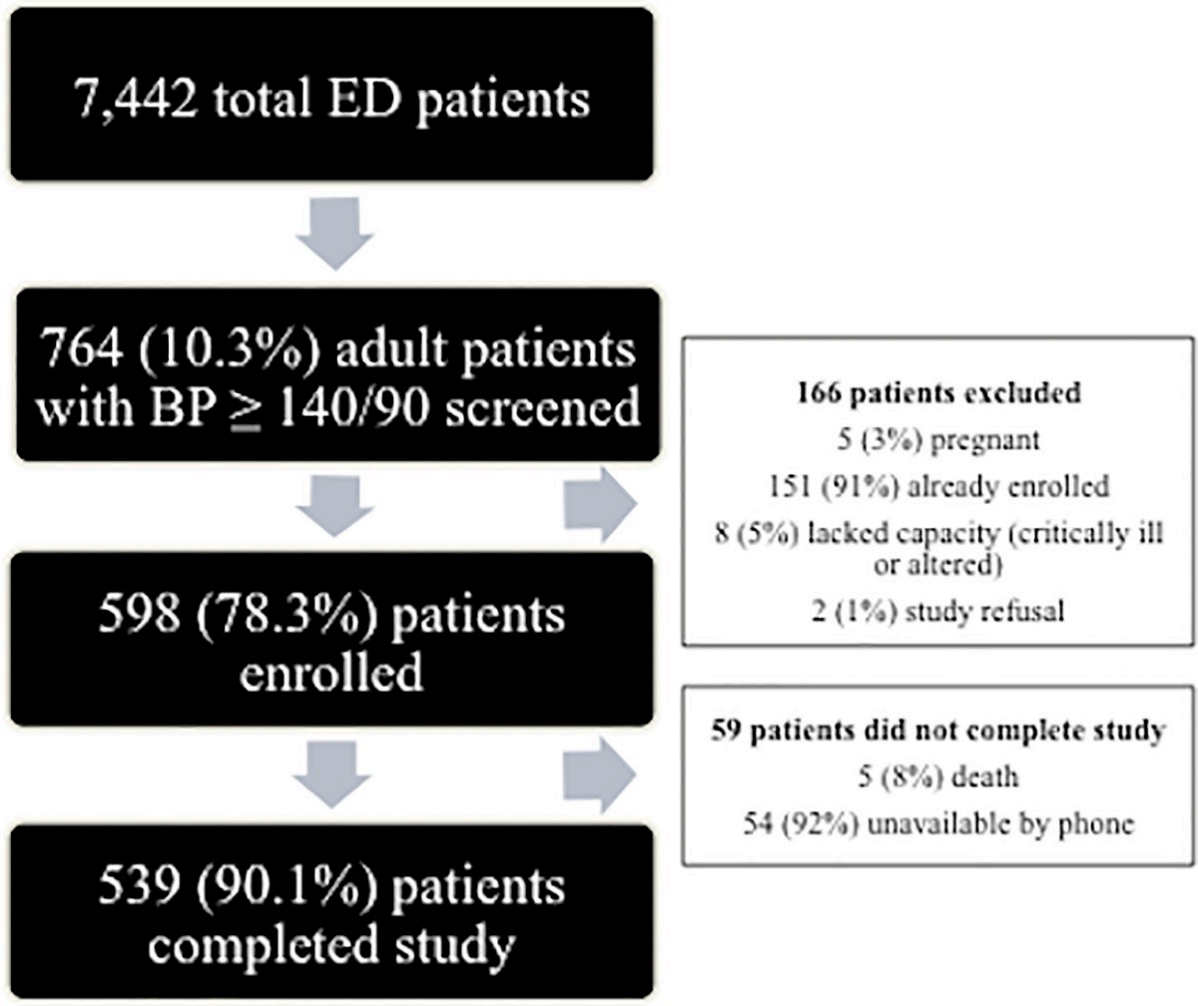
Study enrollment.

From July 2017 to January 2018, 7442 total patients (adults and children) were triaged in the ED during recruitment shifts (8AM -11PM) (Fig 1). Of the 7442 patients, 764 (10.3%) were eligible, of whom 598 (78.3%) enrolled. Of these, 539 (90.1%) completed the study. When comparing individuals who did not complete the study and those who did, no statistically significant (p>0.05) differences in sociodemographic characteristics were observed, including gender, age, ethnicity, alcohol use, or family size. There was a statistically significant (P = 0.02) difference in tobacco use, with 9% of those who did not complete the study currently using tobacco in comparison to 3% use in the study completion cohort.

### Sociodemographics

Of the 539 participants who completed the study, the median age was 61 years (IQR 52–70) (Table 1). The majority were women (n = 337; 62.5%), ethnically Chagga (n = 330; 61.2%), and had a primary school education (n = 309; 57.3%). The most common occupation among participants was farming (n = 331; 61.2%). Many participants reported ongoing use of alcohol (n = 141; 26.2%), while few (n = 17; 3.1%) reported ongoing use of tobacco.

**Table 1:** Characteristics of the study population.

Variable (n, %)	Total (n = 539)
**Gender**	
Male	202 (37.5%)
Female	337 (62.5%)
**Age**	
18–39 years old	33 (6.1%)
40–59 years old	219 (40.6%)
60+ years old	287 (53.3%)
**Ethnicity**	
Chagga	330 (61.2%)
Pare	82 (15.2%)
Sambaa	10 (1.9%)
Other	117 (21.6%)
**Education**	
None	36 (6.7%)
Primary	309 (57.3%)
Secondary	51 (9.5%)
Post-Secondary	143 (26.5%)
**Occupation**	
Unemployed/Retired	29 (5.4%)
Farmer/Wage Earner	331 (61.2%)
Small Business/Vendors	72 (13.4%)
Professional	107 (19.8%)
**Lifestyle Practices**	
Ongoing tobacco use	17 (3.5%)
Ongoing alcohol use	141 (26.2%)

### Hypertension

Of the 539 participants who completed the study, mean systolic blood pressure was 168.8 mmHg (SD 20.8 mmHg) and mean diastolic blood pressure was 100.4 mmHg (SD 12.0 mmHg). All enrolled patients met the study definition of hypertension, and most participants (n = 392, 72.7%) were aware of having a previous diagnosis of hypertension. Most (352; 65.3%) were also currently being treated with anti-hypertensive medication. Nonetheless, 200 patients (37.1%) had uncontrolled hypertension, and 235 (43.6%) participants had evidence of proteinuria, suggestive of microvascular end-organ damage. Among all ED patients (n = 7442), 2423 (32.6%) presented to the ED solely to obtain prescription refills for anti-hypertensive medications.

Of those without a pre-existing diagnosis of hypertension (n = 147), the most common chief complaint was headache (n = 16; 10.9%), followed by abdominal pain (n = 5; 3.4%). In total, 13 patients from this group were admitted to the hospital and the most common admission diagnosis was hypertensive emergency (n = 6; 46.2%). Of those without a pre-existing diagnosis of hypertension (147 patients), the majority (n = 129; 87.8%) reported they were given a diagnosis of hypertension by their outpatient provider at the follow-up visit.

### Follow-up

Only 44% of patients reported follow up with an outpatient medical provider within the month following their ED visit. When comparing individuals who did and did not complete follow-up, no statistically significant (p>0.05) differences in sociodemographic characteristics were observed, including gender, age, ethnicity, or family size (S1 Table). Additionally, we observed no statistically significant (p>0.05) differences in hypertension severity or proteinuria when comparing individuals who did and did not complete follow-up. However, 207 (87.7%) patients who reported follow-up were currently taking anti-hypertensive medications compared to 242 (79.8%) patients who reported no follow-up (P = 0.02). Of the 303 patients that did not follow-up with a medical provider, 16 (5.3%) followed-up with a traditional medicine provider.

Participants reported cost (n = 124; 23%) as a major barrier to follow-up care for their hypertension (Table 2). Participants also reported substantial anxiety related to their diagnosis of hypertension (N = 167; 31%). Participants who reported anxiety about their diagnosis were 20% less likely (RR 0.80; 95% CI 0.64, 0.99) to complete follow-up within 1 month, independent of age, gender, or ethnicity (Table 3).

**Table 2:** Select KAP survey responses among participants with follow-up reported vs. follow-up not reported.

Question (n, %)	Total (n = 539)	Follow-Up Reported (n = 236)	Follow-Up Not Reported (n = 303)	p-value
**Do you think the financial cost of high blood pressure would be a problem for you?**				0.05
Yes	124(23.0%)	45 (19.1%)	79 (26.1%)	
No	415(77.0%)	191(80.9%)	224(73.9%)	
**If you found out you had high blood pressure would you be worried about your future?**				0.04
Yes	167 (31.0)	62 (26.3%)	105(34.7%)	
No	372 (69.0)	174 (73.7%)	198(65.4%)	
**People with high blood pressure may never or rarely feel symptoms?**				0.62
Yes	381 (70.7)	170 (72.0%)	211(69.6%)	
No	58 (10.8.0)	26 (11.0%)	32 (10.6%)	
Unsure/Do Not Know	100 (18.6)	40 (17.0%)	60 (19.8%)	

**Table 3:** Associations between select KAP questions and follow-up.

Variables	Risk Ratios for Follow-Up (95% CI)	
	Unadjusted	Adjusted*
**Worried about the future**	0.79 (0.63, 0.99)	0.80 (0.64, 0.99)
**Understand they must take medication for years**	1.10 (1.01, 1.19)	1.11 (1.03, 1.21)
**Understand hypertension may be asymptomatic**	0.96 (0.86, 1.07)	0.96 (0.86, 1.07)
**Think that financial cost would be a problem**	0.79 (0.61, 1.02)	0.81 (0.63, 1.05)

*Adjusted for age, gender, and ethnicity

## Discussion

In an ED-setting in northern Tanzania, we observed a high burden of elevated blood pressure, with substantial proportions of both uncontrolled hypertension and proteinuria. Additionally, we observed a large proportion of individuals seeking routine prescription refills for anti-hypertensive medications in the ED setting. Despite the high burden, follow-up rates from the ED were low, and anxiety about a future with hypertension was associated with lack of follow-up care.

To our knowledge, this is the first study in SSA to quantify the burden of elevated blood pressure in an ED setting, and it is among the first to explore follow-up care pathways. The alarmingly high proportion (37%) of patients with uncontrolled hypertension is consistent with other studies in Tanzania demonstrating as many as half of all patients diagnosed with hypertension have uncontrolled blood pressure [6, 31–33]. Further, the substantial burden of uncontrolled disease associated with proteinuria in Tanzania is gravely concerning given the increased risk of cardiovascular events and death associated with hypertension and proteinuria [34]. Juxtaposed with the increasing burdens of diabetes, obesity, and alcohol consumption [35, 36], our findings portend that Tanzania is facing an epidemic of cardiovascular morbidity and mortality related to hypertension.

Recent studies have demonstrated that a single hypertensive event in the ED is an independent risk factor for major adverse cardiovascular events [37]. Critical to mitigating that risk is post-ED follow-up [37]. In our study, despite the large proportions of individuals with uncontrolled hypertension, fewer than half followed up with an outpatient provider within one month. As opposed to high-income settings where age and disease severity are predictive of follow-up [38], we found fear about the diagnosis and disease understanding to be important factors associated with follow-up. A recent qualitative analysis from Kenya found major barriers to hypertension care also include the asymptomatic nature of the disease and fear about being a burden to family [39]. These findings are similar to other studies in LMICs, which demonstrate that anxiety about medication side effects and fatalistic attitudes toward diagnoses are associated with lower follow-up rates [40].

The large proportion of individuals using the ED for routine blood pressure treatment was also alarming and higher than utilization rates observed in high-income countries such as the United States (24–29%) and the United Kingdom (18%) [34, 41, 42]. Typically, one ED provider at KCMC is responsible for seeing up to 40 refill patients during an 8-hour shift. During this brief interaction, there is little opportunity for discussion and counseling on lifestyle modifications or dose adjustments, the patients simply receive an updated prescription. Obtaining prescription refills through the emergency department is unsustainable and costly as the majority of patients are paying for ED visits out-of-pocket [43]. Furthermore, district/municipal level insurance plans such as Community Health Fund (CHF) and Tiba Kwa Kadi (TIKA) do not cover care at a regional referral hospitials such as KCMC but would cover outpatient care at municipal level outpatient facilities [11]. Our results suggest that there is an ongoing tremendous unmet need for robust, outpatient hypertension care in Tanzania.

Given the high ED burden of hypertension and the proven benefit of follow-up care, ED settings must take a proactive role in ensuring successful outpatient follow-up. Based on the results of our study, we have identified two potential targets: patient education and reduction of access barriers. Patient education that includes both disease-specific information and general medical literacy may alleviate fears and misconceptions surrounding hypertension [39, 44], while behavior-focused communication strategies may facilitate regular follow-up care [45]. Preliminary studies in high-income settings have shown that brief ED-based educational interventions regarding hypertension improve rates of follow-up and blood pressure control [46, 47]. Though two large-scale randomized, controlled trial comparing behavioral communication strategies, incorporating community health workers in care, and Mhealth in facilitating linkage to care and completion of referrals to providers in Kenya are currently ongoing [48, 49], additional research is needed to determine whether such educational interventions would be beneficial in other resource-limited settings like Tanzania.

In contrast, sustainable and affordable outpatient care is a more complex goal that will likely necessitate the involvement of stakeholders, including government and industry sponsors [33]. Given that there are currently no universal government-sponsored outpatient care plans and less then 20% of the population has insurance coverage, this is a major barrier to overcome. Task-shifting and community microfinance schemes are two potential solutions; there are already effective, local community health worker programs in place for HIV and maternity care that could potentially be adapted for non-communicable diseases [25, 50]. Peer microfinance hypertension programs are another option and have been trialed with success in similar rural settings in Kenya [51]. Given the high economic cost of hypertension-related disability, including stroke and cardiovascular disease, there are major financial incentives to facilitating access to early and affordable hypertension prevention and control strategies on a population level.

Our study had several limitations. Importantly, the time interval for follow-up was brief (one month), and it is unclear if patients continued to be adherent to treatment and care after this period. However, our goal was to determine if providers and patients adhered to international and Tanzanian guidelines for initial follow-up interval (two weeks–one month) [7, 12]. Second, follow-up information for 59 patients (9.9 percent) was missing. This is consistent with previous ED-based studies in this mobile and transient population. Nonetheless, we expect that the patients who were unable to be contacted by our research team were also unlikely to follow-up with an outpatient provider, thus our calculations likely represent an overestimate of follow-up adherence [52]. Additionally, we determined follow-up primarily by patient self-report and only occasionally triangulated this with paper clinic records when there were discrepancies. Thus, social acceptability bias may have resulted in further overestimation of the true follow-up rate. We also did not ask patients where they followed-up (KCMC clinic or community facility) or if they followed-up with a nurse, pharmacist, or physician. In addition, we used proteinuria as a proxy for kidney damage [29]; however, accuracy would have been improved by also checking creatinine levels. We also did not not include self-report of diabetes and obesity in our study or models and these co-morbidities may have influenced follow-up visit results. Finally, some hypertensive patients may have had unusually low blood pressures at time of triage due to acute illness, thus resulting in an underestimation of the true prevalence of hypertension in the ED. Further, we did not include patients with a diagnosis of hypertension who had adequately controlled blood pressure during their ED visit, which likely also resulted in an underestimation of disease prevalence.

In an ED-based sample of adults in northern Tanzania, we observed a high burden of elevated blood pressure, with high rates of uncontrolled disease and end-organ damage. The majority of patients with hypertension did not present for outpatient follow-up care, and both cost and fears about the diagnosis appeared to be major barriers to follow-up care. Novel patient-centered interventions are needed to develop pathways to care to curb the substantial burden of poorly controlled hypertension in the region.

## Supporting information

S1 File Demographic questionnaire. (DOC)

S1 Table Baseline characteristics of participants with follow-up reported vs. follow-up not reported. (DOCX)

S2 Table Associations between all KAP questions and follow-up. *Adjusted for age, gender, and ethnicity.
(DOCX)
