# Supplementary material for: The burden of hypertension in the emergency department and linkage to care: A prospective cohort study in Tanzania
Source: PLoS One. 2019 Jan 25;14(1):e0211287. doi: 10.1371/journal.pone.0211287 (PMC6347227; doi:10.1371/journal.pone.0211287)
Supplement: S1 File — (DOC) [file pone.0211287.s001.doc]

**STUDY ID:** _________________________________

**Today’s Date**: _________________________________

Year Month Day

1. **NAME:** __________________ ________________________

(Given name) (Surname)

1. **Date of Birth (DOB):**  __________ _________ _________ *AND* Age: ________ (yrs)

Year Month Day

1. **Sex**: Female Male ­­
2. **Marital Status**: *(check only one)*

Never Married Currently Married Common law/living with partner

Widowed Separated Divorced

1. **Ethnicity/Tribal Affiliation:** 🞎 Chagga

🞎 Pare

🞎 Maasai

🞎 Sambaa

🞎 Other (specify): ________________________________

1. **Religion/Religious Affiliation**: 🞎 Roman Catholic

🞎 Islam

🞎 Lutheran

🞎 Protestant

🞎 Hindu

🞎 Other (specify): ________________________________

1. **Education Level** (check most appropriate and check highest level only):

**Years:** ____________

🞎 No education (0)

🞎 Primary (1-7 years) (1)

🞎 Secondary (8-11 years) (2)

🞎 High school (12-13 years) (3)

🞎 Trade School/Vocational School (4)

🞎 University/college (5)

1. **Occupation**: _____________________________________________

1. **Household**: _________ Number of Adults living in the house

_________ Number of Children (under 18) in the house

1. **Do you have a Medical History** of High Blood Pressure?

**Yes No**

11. **Do you take medications or herbal remedies for high blood pressure?**

**Yes No**

If yes, specify: _________________________

**12. FEMALE history** *(For Women Only)*:

a. Are you currently pregnant?

**Yes No**

b. Are you currently menstruating?

**Yes NO**

If the female participant answers yes to either of the above questions (12 a. or b.) then he or she is not eligible to participate in our the data collection portion of the study. Please record this an ‘ineligible’ in the Enrollment Logbook.

**13. Tobacco Use**

1. Which best describes your history of tobacco use?

Formerly used Currently Use Never used

tobacco products tobacco products tobacco products

1. At what age did you start? ___________(yrs)

14. Alcohol use

1. Which best describes your history of alcohol use?

Formerly used Currently Use Never used

alcohol products alcohol products alcohol products

1. At what age did you start? ___________(yrs)

15. Why are you seeking care at KCMC? (Chief complaint?) -__________________________

**Data Collection**

**16. Triage Blood Pressure**: _________________________________

**17. 60 – 90min Blood Pressure**: ___________ ___________

1^st^ 2^nd^

**18. Urinalysis: Is protein present?**

**YES NO**

**Disposition**

**19. Admitted to the hospital?**

**Yes NO**

**20. If Yes, Admission diagnosis _____________**

**21. If discharged, are they given a follow-up appointment?**

**Yes NO**

**22. If discharged, are blood pressure medications added or changed? Specify**

**_______________________**
