# Supplementary material for: The burden of hypertension in the emergency department and linkage to care: A prospective cohort study in Tanzania
Source: PLoS One. 2019 Jan 25;14(1):e0211287. doi: 10.1371/journal.pone.0211287 (PMC6347227; doi:10.1371/journal.pone.0211287)
Supplement: S1 Table — (DOCX) [file pone.0211287.s002.docx]

**Supplemental Table 1. Baseline characteristics of participants with follow-up reported vs. follow-up not reported.**

| **Variable (n, %)** | **Total(N=539)** | **Follow-Up Reported (n=236)** | **Follow-Up Not Reported (n=303)** | ***p*** |
| --- | --- | --- | --- | --- |
| **Gender** |  |  |  | 0.25 |
| Male | 202 (37.5%) | 82 (34.7%) | 120 (39.6%) |  |
| Female | 337 (62.5%) | 154 (65.3%) | 183 (60.4%) |  |
| **Age** |  |  |  | 0.26 |
| 18–39 years old | 33 (6.1%) | 10 (4.2%) | 23 (7.6%) |  |
| 40–59 years old | 219 (40.6%) | 96 (40.7%) | 123 (40.6%) |  |
| 60+ years old | 287 (53.3%) | 130 (55.1.5%) | 157 (51.8%) |  |
| **Ethnicity** |  |  |  | 0.72 |
| Chagga | 330 (61.3%) | 151 (64.0%) | 179 (59.1%) |  |
| Pare | 82 (15.2%) | 33 (14.0%) | 49 (16.2%) |  |
| Sambaa | 10 (1.9%) | 4 (1.7%) | 6 (2.0%) |  |
| Other | 117 (21.6%) | 48 (20.3%) | 69 (22.8%) |  |
| **Education** |  |  |  | 0.15 |
| None | 36 (6.7%) | 21 (8.9%) | 15 (5.0%) |  |
| Primary | 309 (57.3%) | 139 (58.9%) | 170 (56.1%) |  |
| Secondary | 51 (9.5%) | 22 (9.3%) | 29 (9.6%) |  |
| Post-Secondary | 143 (26.5%) | 54 (22.9%) | 89 (29.4%) |  |
| **Occupation** |  |  |  | 0.68 |
| Unemployed/Retired | 29 (5.4%) | 19 (4.2%) | 10 (12.8%) |  |
| Farmer/Wage Earner | 331 (61.2%) | 146 (61.9%) | 185 (61.0%) |  |
| Small Business/Vendors | 72 (13.4%) | 31 (13.6%) | 40 (13.2%) |  |
| Professional | 107 (19.8%) | 48 (20.3%) | 59 (19.5%) |  |
| **Lifestyle Practices** |  |  |  |  |
| Ongoing tobacco use | 17 (3.5%) | 5 (2.12%) | 12 (4.0%) | 0.22 |
| Ongoing alcohol use | 141 (26.2%) | 59 (25.0%) | 82 (27.0%) | 0.59 |
| **Total Household Members** |  |  |  |  |
| > 2 Children | 114 (21.2%) | 46 (19.5%) | 68 (22.4%) | 0.41 |
| > 4 Adults | 81 (15.1%) | 38 (16.0%) | 43 (14.2%) | 0.53 |
