# Supplementary material for: The burden of hypertension in the emergency department and linkage to care: A prospective cohort study in Tanzania
Source: PLoS One. 2019 Jan 25;14(1):e0211287. doi: 10.1371/journal.pone.0211287 (PMC6347227; doi:10.1371/journal.pone.0211287)
Supplement: S2 Table — *Adjusted for age, gender, and ethnicity. (DOCX) [file pone.0211287.s003.docx]

**Supplemental Table 2. Associations between all KAP questions and follow-up.**

| **KAP Question** | **Risk Ratios for Follow-Up (95% CI)** | |
| --- | --- | --- |
|  | **Unadjusted** | **Adjusted*** |
| **Do you think high blood pressure might cause kidney disease** | 0.94 (0.87, 1.01) | 0.94 (0.87, 1.02) |
| **Do you think that diets rich in salt can cause high blood pressure?** | 1.02 (0.90, 1.14) | 1.03 (0.92, 1.17) |
| **Would you be worried about your reputation in the community if you found out that you have high blood pressure?** | 1.10 (0.80, 1.51) | 1.10 (0.80, 1.51) |
| **Would you be worried about your ability to work if you found out that you have high blood pressure?** | 0.85 (0.67, 1.06) | 0.85 (0.67, 1.08) |
| **Would you be worried about your chances of survival if you found out that you have high blood pressure?** | 1.04 (0.80, 1.34) | 1.02 (0.79, 1.32) |
| **Do you think that high blood pressure is a problem in Kilimanjaro?** | 0.85 (0.70, 1.02) | 0.84 (0.69, 1.01) |
| **Do you think that the cost of high blood pressure would be a problem for you?** | 0.79 (0.61, 1.02) | 0.81 (0.63, 1.05) |
| **How likely would you be to seek care from a traditional healer?** | 0.97 (0.82, 1.16) | 0.98 (0.82, 1.17) |
| **How likely would you be to seek self-treatment at home?** | 1.03 (0.91, 1.16) | 1.02 (0.91, 1.15) |
| **How likely would you be to seek care at a hospital or health clinic?** | 0.97 (0.83, 1.15) | 0.96 (0.82, 1.13) |
| **Would you be willing to be contacted by cell phone regarding care of your high blood pressure?** | 0.96 (0.84, 1.09) | 0.97 (0.85, 1.10) |
| **Would you be willing to be contacted by text message regarding care of your high blood pressure?** | 0.91 (0.80, 1.04) | 0.92 (0.81, 1.04) |
| **Herbal or natural medications are commonly used to treat health problems. Herbal or natural medications may include herbs, teas, foods, creams, lotions, potions, and soups that are used to treat health problems. How likely would you be to use herbal or natural medications if you found out that you have high blood pressure?** | 0.99 (0.88, 1.12) | 0.99 (0.88, 1.11) |
| **How likely would you be willing to see a Medical Doctor if you found out that you have high blood pressure?** | 0.91 (0.75, 1.10) | 0.91 (0.74, 1.10) |

*Adjusted for age, gender, and ethnicity
